# COX6A2 deficiency leads to cardiac remodeling in human pluripotent stem cell-derived cardiomyocytes

**DOI:** 10.1186/s13287-023-03596-x

**Published:** 2023-12-10

**Authors:** Mengqi Jiang, Yuanxiu Song, Xi Chen, Wenjing Lu, Min Zhu, Mingyu Wei, Feng Lan, Ming Cui, Yun Bai

**Affiliations:** 1 Department of Cell Biology, School of Basic Medical Sciences, Peking University Health Science Center, Beijing, 100191 China; 2 Department of Cardiology, Peking University Third Hospital, 49 Huayuan North Road, Haidian District, Beijing, 100191 China; 3 Shenzhen Key Laboratory of Cardiovascular Disease, Fuwai Hospital Chinese Academy of Medical Sciences, Chinese Academy of Medical Sciences and Peking Union Medical College, Shenzhen, 518057 China; 4 State Key Laboratory of Cardiovascular Disease, Fuwai Hospital, National Center for Cardiovascular Diseases, Chinese Academy of Medical Sciences and Peking Union Medical College, Beijing, 100037 China

**Keywords:** Human cardiomyocyte, Cardiac remodeling, COX6A2, hiPSCs, CRISPR/Cas9, Oxidative stress, Drug discovery

## Abstract

**Background:**

Cardiac remodeling is the initiating factor for the development of heart failure, which can result from various cardiomyopathies. Cytochrome c oxidase subunit 6A2 (COX6A2) is one of the components of cytochrome c oxidase that drives oxidative phosphorylation. The pathogenesis of myocardial remodeling caused by COX6A2 deficiency in humans remains unclear because there are no suitable research models. In this study, we established a COX6A2-deficient human cardiac myocyte (CM) model that mimics the human COX6A2 homozygous mutation and determined the effects of COX6A2 dysfunction and its underlying mechanism.

**Methods:**

A human COX6A2 homozygous knockout cardiomyocyte model was established by combining CRISPR/Cas9 gene editing technology and hiPSC-directed differentiation technology. Cell model phenotypic assays were done to characterize the pathological features of the resulting COX6A2-deficient cardiomyocytes.

**Results:**

COX6A2 gene knockout did not affect the pluripotency and differentiation efficiency of hiPSCs. Myocardial cells with a COX6A2 gene knockout showed abnormal energy metabolism, increased oxidative stress levels, abnormal calcium transport activity, and decreased contractility. In addition, L-carnitine and trimetazidine significantly improved energy metabolism in the COX6A2-deficient human myocardial model.

**Conclusions:**

We have established a COX6A2-deficient human cardiomyocyte model that exhibits abnormal energy metabolism, elevated oxidative stress levels, abnormal calcium transport, and reduced contractility. This model represents an important tool to gain insight into the mechanism of action of energy metabolism disorders resulting in myocardial remodeling, elucidate the gene-phenotype relationship of COX6A2 deficiency, and facilitate drug screening.

**Supplementary Information:**

The online version contains supplementary material available at 10.1186/s13287-023-03596-x.

## Background

Cardiac remodeling refers to the pathological state of abnormal changes in cardiac structure, metabolism, and function caused by myocardial damage and/or the increased load by stimulation with various pathogenic factors, which is the initial factor of heart failure. At the molecular level, cardiac remodeling may be specifically manifested as cardiac chamber enlargement, apoptosis, excessive deposition, increased degradation of myocardial extracellular matrix, and fibrosis replacement; however, the specific mechanism is unclear [1]. Although there are differences in the mechanisms of cardiac remodeling caused by different etiologies, the energy metabolism disorder in cardiomyocytes is the final common pathway of cardiac remodeling [2]. Myocardial fibrosis occurs during the process of myocardial remodeling. It affects the function of cardiomyocytes, and type I collagen α1 chain (COL1A1) and type IV collagen α1 chain (COL4A1) play an important role in inhibiting the occurrence and development of myocardial fibrosis [3]. Myosin heavy chain α (MYH6) and myosin heavy chain β (MYH7) are two important subunits of myosin [4] that play an important role in maintaining the normal myofilaments and sarcomere structure and function of cardiomyocytes.

Mitochondria represent the primary site of myocardial energy production and metabolism, accounting for 30% of the total volume of myocardial cells [5] and the energy produced can account for 90% of the total energy [6, 7]. The heart is the largest energy-consuming organ in the human body and is a high energy-demand tissue. Mitochondria, as the main organelle for energy production, accounts for 30% of the total volume of cardiomyocytes [8]. Therefore, maintaining the integrity and function of mitochondria is important for maintaining the myocardium's normal structure and physiological function.

Respiratory chain complex IV (cytochrome C oxidase, COX), which is the terminal enzyme complex of the mitochondrial electron transport chain, catalyzes the transfer of electrons from reduced cytochrome c to molecular oxygen and the transfer of protons to the inner mitochondrial membrane. The establishment of this electrochemical gradient across the membrane provides the driving force for ATP synthesis [9]. The COX6A protein is one of 13 subunits in complex IV of the respiratory chain; however, its precise role in this complex is unknown. Possible functions include the assembly of the complex as well as the regulation of the catalytic activity of the core subunit. Mammalian COX6A consists of two distinct isoforms, Cox6a-L (COX6A1, hepatic type) and COX6A-H (COX6A2, cardiac type). The COX6A1 subunit is widely expressed, whereas COX6A2 expression is restricted to striated muscle [10, 11]; however, recent studies have found that COX6A2 is also present in nerve cells. Thus far, functional studies of COX6A2 are sparse. A COX6A2 gene knockout mouse model exhibited diastolic cardiac dysfunction when the cardiac load was increased [12], showed a protective effect on insulin resistance [11], and COX6A2 protected neurons from oxidative stress [13]. Concerning case reports, two patients harboring a COX6A2 mutation presented with limb muscle weakness, hypotonia, and facial muscle weakness, whereas one of them had cardiomyopathy [14]. This suggests that COX6A2 has an important role in cardiomyocytes. Thus, our study aimed to determine the role of COX6A2 in maintaining the normal function of cardiomyocytes.

## Methods

### hiPSC culture and cardiac differentiation

The U2 human induced pluripotent stem cell line (hiPSCs-U2, purchased from Cellapy, China) was cultured in 6-well plates (Corning, USA) coated with Matrigel (Corning, USA), and the medium was changed each day. When the cell confluence reached 80–90%, it was replaced with myocardial differentiation medium (Cellapy, China) consisting of just three components: the basal medium RPMI 1640, L-ascorbic acid 2-phosphate and rice-derived recombinant human albumin [15]. On the 10th day of differentiation, the cells that could beat autonomously appeared.

### Genome editing

The COX6A2 single-stranded guide RNA (sgRNA) (5′-GTGGCCTCCTTTGGCAGCGC-3′) was designed using an online tool (https://design.synthego.com). The epiCRISPR vector was introduced along with sgRNA into hiPSCs by electroporation using the 4D nuclear receptor system (Lonza, Germany) and the CA137 program. The treated cells were re-seeded into 6-well plates and drug screening (puromycin) was initiated when the cells reached 40% confluence to obtain homozygous transfected strains.

### Immunofluorescent staining

Cells were seeded on Matrigel-coated 20 mm coverslips and fixed with 4% PFA for 15 min. The cell membranes were disrupted with 0.2% Triton X-100 (Sigma, USA) and blocked with 3% BSA (Sigma, USA) for 30 min. Primary antibody was added and incubated at 4 °C overnight followed by incubation with secondary antibody (Invitrogen, USA) at 37 °C for 1 h in the dark. The cells were washed with PBS and fixed in fluorescence-blocking tablets containing DAPI (4, 6-diamino-2-phenylindole). Images were taken under a confocal microscope (Leica DMI 4000B, Germany). Antibodies and their working dilutions are listed in Additional file 1: Table S1.

### Western blot analysis

Cells were trypsin-digested, centrifuged, and lysed using a protein extraction reagent (Thermo Fisher, USA) supplemented with a mixture of phosphatase and protease inhibitors (Thermo Fisher, USA). The entire cleavage process was carried out on the ice and the samples were shaken every 10 min with an oscillator, centrifuged at 12,000 rpm for 15 min, and the supernatant was collected as the protein sample. Protein concentration was determined by the BCA method. For gel electrophoresis, equal amounts of protein were loaded onto a polyacrylamide gel, electrophoresed, and transferred to a polyvinylidene fluoride (PVDF) membrane. The membranes were blocked with 5% skim milk powder for 1 h at 37 °C and incubated with the primary and secondary antibodies listed in Additional file 1: Table S2.

### Flow cytometry

The detected cardiomyocytes were treated with CardioEasy Human Cardiomyocyte Digestive Fluid (Cellapy, China) to form a single-cell suspension. After washing with PBS, the cells were fixed (except for viable cell assays), stained with various antibodies, re-filtered through a 300-mesh filter, and analyzed immediately by FACS (Beckman, USA). The number of cells examined in one group was not less than 1 million. The results were analyzed by the Flow Jo program.

### RNA extraction and quantitative real-time PCR

Total RNA was extracted from cells using TRIZOL reagent (Invitrogen, USA) and reverse-transcribed into cDNA using the Prime Script Reverse Transcription system (Takara, Japan). Quantitative RT-PCR was done using SYBR Green II (Takara, Japan) and the iQ5 instrument (Bio-Rad, Hercules, CA). A comparative CT method was used to analyze the relative changes in gene expression. The results are expressed relative to the expression of β-actin (internal control). Primer sequences are listed in Additional file 1: Table S2.

### RNA sequencing (RNA-Seq) assay

The mRNA library was established with purified cardiomyocytes on day 45 of differentiation. Total RNA was extracted from cardiomyocytes and purified by RNase H and DNase I. The cDNA was then synthesized. The double-stranded DNA was purified by magnetic beads and amplified by PCR. The PCR products were denatured and cycled into single-stranded circular DNA (ssCir DNA), which was further amplified to become the final library. Qualified libraries were analyzed by the BGISEQ500 platform, single-ended 50-base reads (SE50). Clean reads were obtained after pruning reads with low quality, contamination, or high levels of unknown bases. These clean reads were then compared to reference genome sequences using Bowtie2 and HISAT2. More significant and credible differentially expressed genes (DEGs) were screened out based on |log2 Fold change|≥ 1 and adjusted *P* value (FDR, *Q*-value) < 0.001. Gene Ontology and KEGG pathway enrichment analysis of these DEGs were performed by Phyper under the Hypergeometric test (https://en.wikipedia.org/wiki/Hypergeometric_distribution). The *Q*-value of terms or pathways less than 0.05 was significantly enriched.

### Transmission electron microscopy

The detected cardiomyocytes were treated with CardioEasy Human Cardiomyocyte Digestive Fluid (Cellapy, China). The cell suspension was centrifuged and fixed with 0.10% GA + 4% PFA. After dehydration, the samples were prepared and subject to transmission electron microscopy (Thermo, USA) for image acquisition.

### Seahorse assay

The cells were seeded into a cell culture plate and the Seahorse XFe24 Flux Assay Kit was used. Then, the Agilent Seahorse XFe24 system was used to detect the respiratory and reserve functions of the cells.

### Ca^2+^ imaging

Cardiomyocytes were seeded and incubated with Fluo-4 (Beyotime, China). The spontaneous calcium transient signals of cardiomyocytes were collected by confocal microscopy (Leica, USA) at a high frame rate. The results were analyzed by ImageJ and IGOR Pro.

### Detection of myocardial contractility

The cells were cultured in 6-well plates (Corning, USA) coated with Matrigel (Corning, USA). The video was captured using a Leica DMI 4000B. Videos of cardiomyocyte beats were taken for 3–5 s, saved in the original.czi format, and converted to an uncompressed.avi format [70 frames per second (fps)]. A special plugin for video analysis (MUSCLEMOTION) was installed in ImageJ to analyze the results [16].

### Detection of cellular ATP content

Lysate (200 µl) was added to each well of a 6-well plate, and the supernatant was collected after cell lysis. The cells were then examined using an ATP detection kit (Beyotime, China), and the relative light unit values were measured with a luminometer or liquid scintillation meter to measure the ATP content in the cells. The concentration of ATP in the sample was calculated from standard curves and cell numbers.

### Data analysis and statistics

Results are expressed as the mean ± SD. Statistical analysis was performed with GraphPad Prism 8.0.1 for Windows. A two-sided unpaired Student’s *t* test was used to compare two groups with a normal distribution. A one-way ANOVA was used to compare three or more groups. All tests for normality and homogeneity of variance were passed before administering the *t* test and one-way analysis of variance. *P* values of less than 0.05 were considered statistically significant (*P < 0.05, **P < 0.01, ***P < 0.001; ns, not significant).

## Results

### Establishing a COX6A2-deficient hiPSC model

**Fig. 1:**
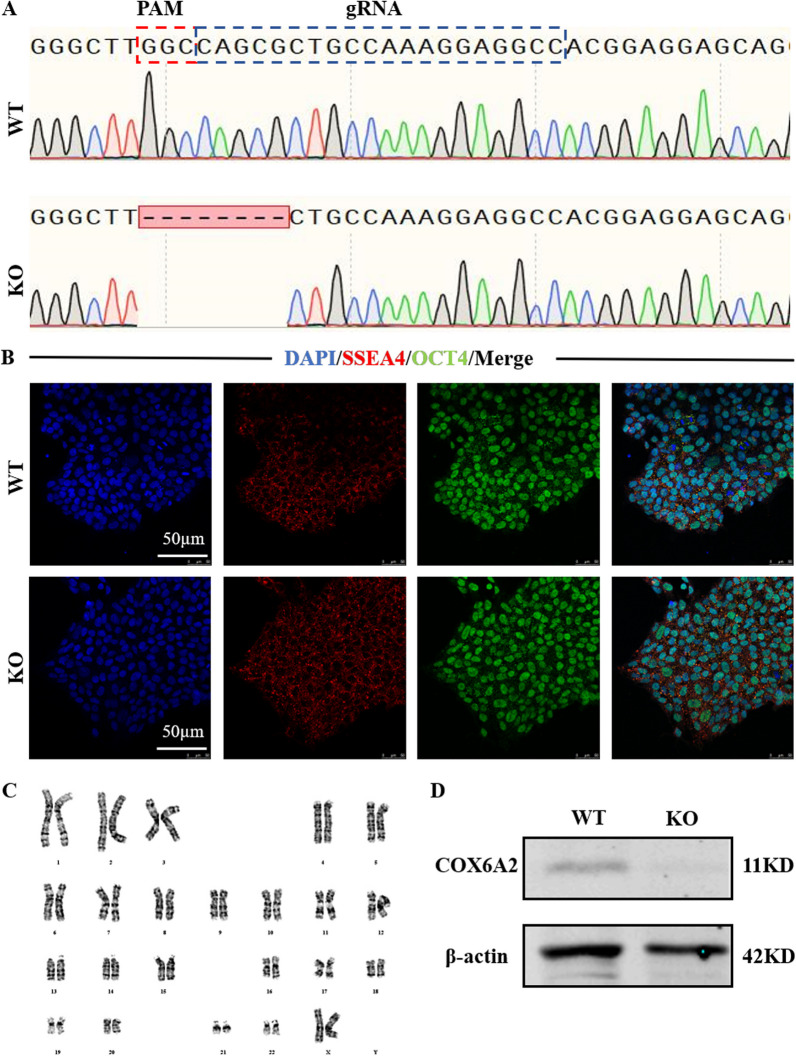
Establishment of COX6A2^−/−^hiPSCs. **A** Diagram of the COX6A2 knockout pattern, showing the gene editing position and deleted base pair. **B** Immunofluorescence staining of COX6A2^−/−^hiPSCs showed that COX6A2^−/−^hiPSCs expressed OCT4 and SSEA4 normally, scale bar = 50 µm. **C** The karyotype analysis of KO-hiPSCs showed that KO-hiPSCs had normal female chromosomes. **D** The expression level of COX6A2 protein was detected by Western Blot

We established a COX6A2-deficient hiPSC cell model using the CRISPR/Cas9 system. We designed sgRNA targeting the COX6A2 gene in cells and constructed plasmids containing sgRNA and Cas9 elements. The epiCRISPR vector along with sgRNA was introduced into hiPSCs by electroporation, and the cells were screened with puromycin. The genotypes of the surviving clones were determined by Sanger sequencing. The results showed that homozygous COX6A2-knockout hiPSCs (COX6A2^−/−^hiPSCs) were successfully obtained (Fig. 1A). COX6A2^−/−^hiPSCs grew as clonogenic cell clusters as normal hiPSCs (Additional file 1: Fig. S1A). Immunofluorescence staining of the stem cell markers, OCT4 and SSEA4, was performed on the resulting cell lines, and the results indicated that COX6A2 knockout did not affect the expression of stemness markers in hiPSCs (Fig. 1B). Next, we carried out karyotype detection on COX6A2^−/−^hiPSCs, which indicated that COX6A2^−/−^hiPSCs had maintained a normal karyotype (Fig. 1C). Furthermore, teratoma assays revealed that COX6A2^−/−^hiPSCs preserved the ability to differentiate into three germ layers (Figure S1B). To verify the successful knockdown of COX6A2 at the protein level, western blot analysis was done. The results indicated that no significant COX6A2 protein expression was present in COX6A2^−/−^hiPSCs (Fig. 1D, full-length blots, and the quantification data of the WB result are presented in Additional file 1: Fig. S2).

**Fig. 2:**
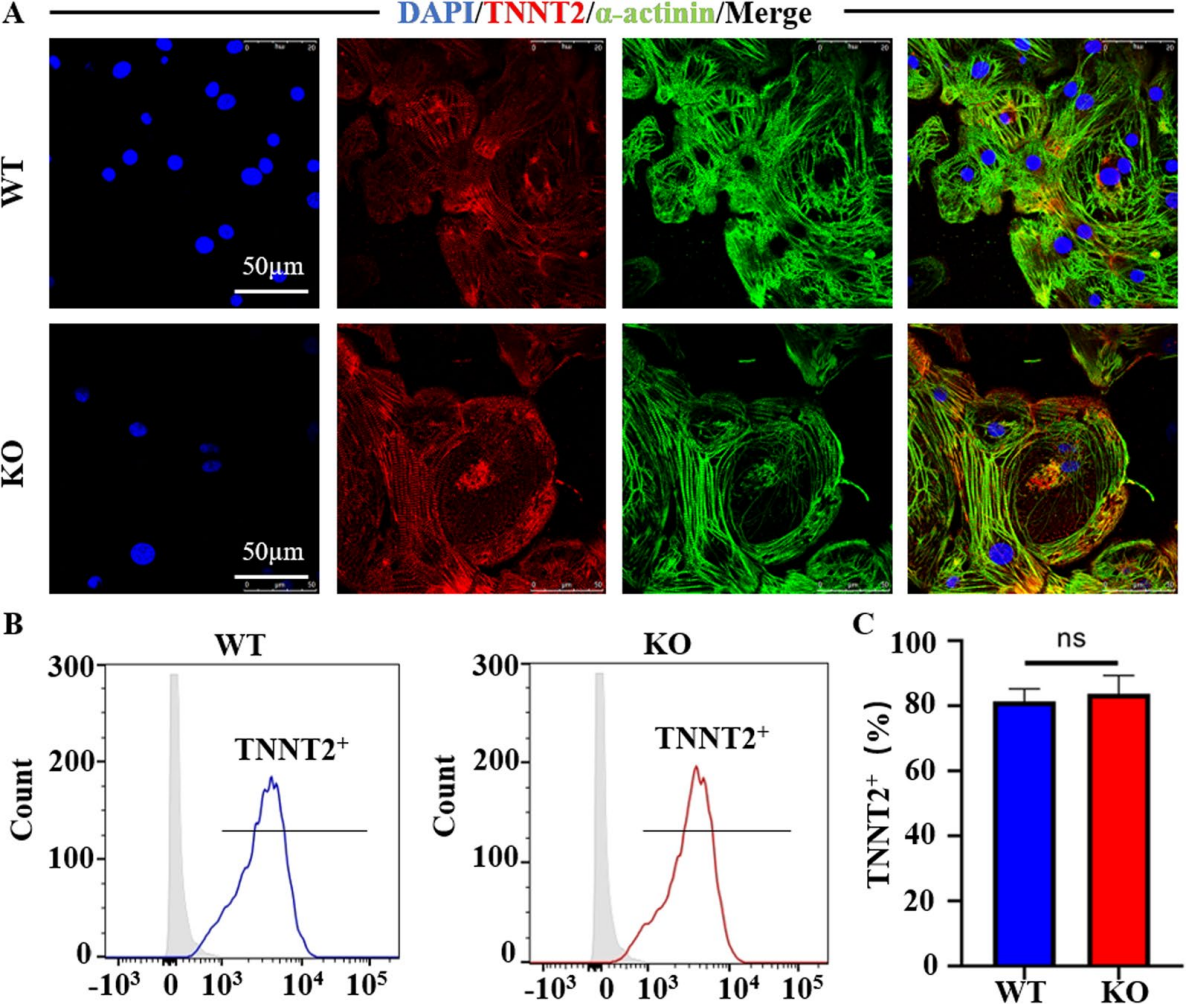
COX6A2 knockout did not affect the myocardial differentiation of hiPSCs. **A** COX6A2^−/−^hiPSC-CMs normally expressed TNNT2 and α-actinin, and the myofilament was arranged neatly. **B** Flow cytometry was used to detect the positive rate of TNNT2 in unpurified WT and KO cardiomyocytes on day 10. **C** The quantitative statistical plot of the differentiation efficiency of WT and KO hiPSC-CMs. The results are presented as mean ± SD of 3 independent experiments. ns, not significant

### COX6A2^−/−^hiPSCs showed normal myocardial differentiation

To verify whether COX6A2 gene knockdown affects myocardial differentiation, we differentiated hiPSCs and COX6A2^−/−^hiPSCs into cardiomyocytes and observed their differentiation efficiency. This differentiation protocol can produce TNNT2^+^ cardiomyocytes efficiently. We successfully induced hiPSCs and COX6A2^−/−^hiPSCs to differentiate into cardiac myocytes, hiPSC-CMs (WT), and COX6A2^−/−^hiPSC-CMs (KO), respectively. The spontaneous and regular beating of the reticular myocardium was observed from days 9 to 10 of differentiation.

To determine the effect of COX6A2 knockdown on the differentiation of hiPSCs into hiPSC-CMs, the differentiation efficiency of cardiomyocytes in WT and KO groups was evaluated. TNNT2 is a protein specifically expressed in cardiomyocytes and is often used as a marker for cardiomyocyte detection [15]. TNNT2 and α-actinin are commonly used markers to detect myofilaments and the sarcomere structure of cardiomyocytes [17]. Immunofluorescence staining was used to detect TNNT2 and α-actinin in the two cardiomyocyte groups and the results indicated that TNNT2 and α-actinin were normally expressed. The myofilaments and sarcomere arrangement in KO cardiomyocytes were also very regular and clear, which preliminarily indicated that cardiomyocytes in both groups differentiated normally (Fig. 2A). To further verify whether the differentiation efficiency of the two groups of cardiomyocytes was consistent, we used flow cytometry to detect TNNT2 expression on both types of cardiomyocytes cultured without purification on the 10th day of differentiation. The results indicated that the expression ratio of TNNT2 in both WT and KO groups was approximately 80% and there was no statistical difference (Fig. 2B, C). This indicated that the COX6A2 gene knockout human cardiomyocyte model was successfully established and COX6A2 deletion did not affect the differentiation efficiency of the cardiomyocytes.

### COX6A2^−/−^hiPSC-CMs showed myocardial remodeling phenotype

**Fig. 3:**
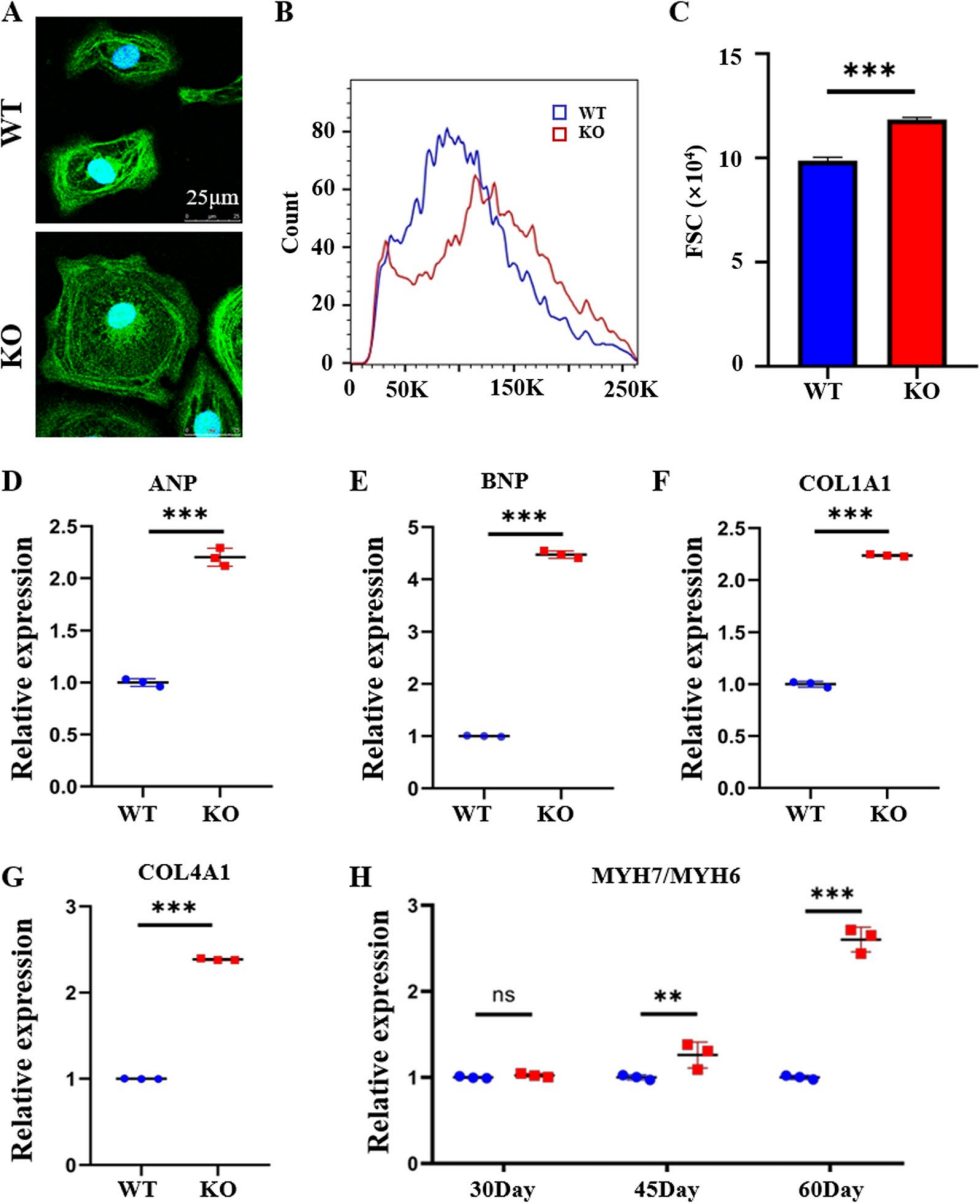
KO hiPSC-CMs showed increased cell size and a myocardial remodeling phenotype. **A** The size of WT and KO hiPSC-CMs was detected by phalloidin staining, scale bar = 25 µm. **B** FSC values measured by flow cytometry were used to compare the sizes of WT and KO hiPSC-CMs. **C** Quantitative statistical plots of FSC values in WT and KO hiPSC-CMs. **D**, **E**, **F**, **G**, **I** The expression levels of ANP, BNP, COL1A1, and COL4A1 in WT and KO hiPSC-CMs were detected by qPCR. **H** The expression level of MYH7/MYH6 in WT and KO hiPSC-CMs at 30, 45, and 60 days was detected by qPCR. The results are presented as mean ± SD of 3 independent experiments. ns, not significant; **P < 0.01; ***P < 0.001

Cardiac hypertrophy and myocardial remodeling occur in cardiomyocytes under pathological conditions, which are the initial factors leading to heart failure. To determine the effect of COX6A2 deletion on cardiomyocyte morphology, we first examined the size of COX6A2^−/−^hiPSC-CMs. The two types of myocardium developed to 40 days were inoculated as cell crawling slices. The cardiomyocyte cytoskeleton was stained with phalloidin and the surface area of the KO cardiomyocytes was significantly increased compared with the WT cardiomyocytes as determined by laser confocal microscopy (Fig. 3A). Flow cytometry was used to evaluate myocardial cell volume and the results showed that compared with WT myocardial cells, the forward scattering angle (FSC) or the KO myocardial cells appearing as a metric peak moved to the right (Fig. 3B, C). This further verified that the COX6A2-KO myocardial cell size significantly increased, presenting hypertrophy myocardial morphology. Next, we examined the cardiac hypertrophy and heart failure-related markers, atrial natriuretic factor (ANP), and brain natriuretic peptide (BNP) in KO cardiomyocytes [18]. Using qPCR, we found that the expression of ANP, BNP, COL1A1, and COL4A1 mRNA in cardiomyocytes following COX6A2 knockdown was significantly increased compared with that of the WT cells (Fig. 3D–G), suggesting that the cardiomyocytes developed toward cardiac hypertrophy and fibrosis. We further measured the MYH7/MYH6 ratio of WT and KO hiPSC-CMs on days 30, 45, and 60 of differentiation. The results showed that with the continuous maturation of cardiomyocytes, the MYH7/MYH6 ratio of KO hiPSC-CMs was gradually increased compared with WT hiPSC-CMs, and KO hiPSC-CMs showed a pathological phenotype of myocardial hypertrophy (Fig. 3H). This phenotype is consistent with the pathological characteristics of DCM, which further suggests that COX6A2 deletion leads to myocardial remodeling phenotypes in cardiomyocytes.

### Mitochondrial morphology and function are affected in COX6A2-deficient cardiomyocytes

The normal form of mitochondria is important for maintaining physiological function. Changes in mitochondrial function also affect mitochondrial morphology [19, 20]. To determine the effects of oxidative stress on mitochondrial function in cardiomyocytes caused by COX6A2 knockdown, mitochondrial function was assessed.

**Fig. 4:**
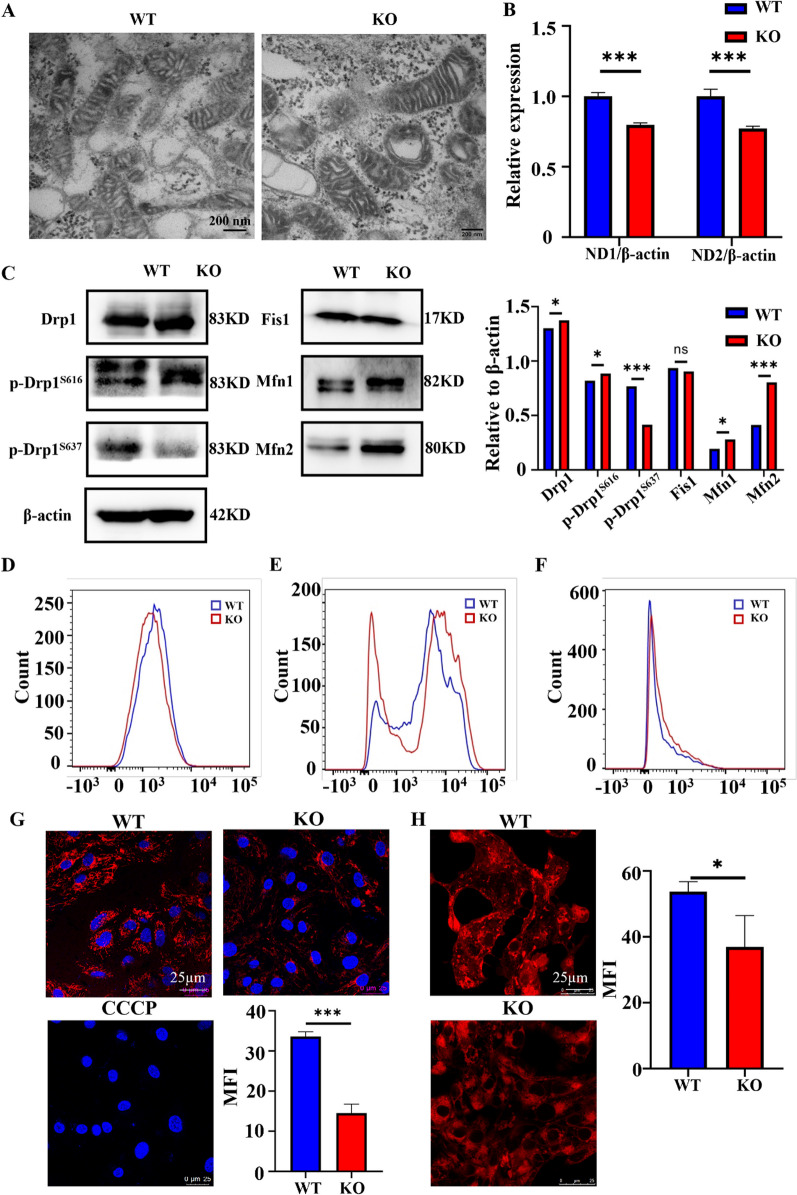
KO hiPSC-CMs showed abnormal mitochondrial function and elevated oxidative stress levels. **A** The mitochondrial morphology of WT and KO hiPSC-CMs was detected by transmission electron microscopy, scale bar = 200 nm. **B** The expression levels of ND1 and ND2 in WT and KO hiPSC-CMs were detected by qPCR, and the expression of ND1 and ND2 was down-regulated in KO cardiomyocytes, suggesting that mitochondrial DNA was reduced in KO hiPSC-CMs. **C** The expression level of Drp1, p-Drp1^S616^, p-Drp1^S637^, Fis1, Mfn1, and Mfn2 protein was detected by Western Blot, and the quantification data of the WB result. **D** Flow cytometry (Mitotracker labeling) showed the average fluorescence intensity distribution of mitochondria in WT and KO hiPSC-CMs. **E** The mean fluorescence intensity distribution of mitochondrial ROS in WT and KO hiPSC-CMs was detected by flow cytometry. **F** Flow cytometry (MitoSOX Red labeling) showed the mean fluorescence intensity distribution of mitochondrial ROS in WT and KO hiPSC-CMs. **G** The mitochondrial membrane potential levels of WT and KO hiPSC-CMs were detected by immunofluorescence staining (TMRE labeling), scale bar = 25 µm. **H** The representative images of Mitotracker staining of WT and KO hiPSC-CMs were detected by immunofluorescence staining and the statistical results also showed, scale bar = 25 µm. The results are presented as mean ± SD of 3 independent experiments. ns, not significant; *P < 0.05, ***P < 0.001

First, we used transmission electron microscopy to visually observe the morphology of mitochondria in the WT and KO cardiomyocyte groups. Compared with WT cardiomyocytes, the size of the mitochondria in the KO cardiomyocytes was somewhat enlarged and the mitochondrial crista was relatively reduced (Fig. 4A, Additional file 1: Fig. S3A). We also examined the mitochondrial DNA content of the two cardiomyocyte groups to compare the number of mitochondria. Mitochondria encodes NADH Dehydrogenase 1 and 2 (ND1, ND2), which are proteins encoded by mitochondrial DNA and reflect mitochondrial number [21]. Quantitative PCR was used to measure ND1 and ND2 content in the cardiomyocytes of both groups. The expressions of ND1 and ND2 in KO cardiomyocytes were significantly lower compared with that in the WT group (Fig. 4B), indicating that COX6A2 knockout resulted in a decrease in the number of mitochondria in cardiomyocytes. To confirm this result, we further detected the protein expression levels of Drp1, p-Drp1S616, p-Drp1S637, Fis1, Mfn1, and Mfn2 in WT and KO hiPSC-CMs (Fig. 4C, full-length blots of the WB result are presented in Additional file 1: Fig. S4). The results showed that the expression of the genes that promote mitochondrial fusion increased in COX6A2 KO hiPSC-CMs, while the genes that inhibit mitochondrial fusion decreased. This was consistent with the results of previous electron microscopy experiments, which further indicated that COX6A2 knockout could cause changes in the mitochondrial morphology of cardiomyocytes.

Next, we used the mitochondrial probe Mitotracker to detect the number of mitochondria in WT and KO cardiomyocytes by flow cytometry and immunofluorescence staining techniques and found that the average fluorescence intensity of KO cardiomyocytes was significantly lower than that of WT cardiomyocytes (Fig. 4D, H and Additional file 1: Fig. S3B). These results indicate that COX6A2 knockdown resulted in a decrease in the number of mitochondria.

Reactive oxygen species (ROS) are produced during cardiomyocyte aerobic metabolism. Approximately 10% of the ROS is produced during normal cellular metabolism and excessive intracellular ROS can cause damage to biological macromolecules, such as lipids, proteins, and nucleic acids [22]. Oxidative stress injury caused by ROS and subsequent apoptosis of cardiomyocytes is a major cause of cardiomyopathy-related pathological phenotypes as well as heart failure [23]. DCFH-DA was used to detect ROS levels in WT and KO cardiomyocytes and the fluorescence intensity of the KO cardiomyocytes was significantly decreased compared with the WT cardiomyocytes (Fig. 4E and Additional file 1: Fig. S3C), indicating that the ROS levels in KO cardiomyocytes were significantly higher compared with that of WT cardiomyocytes.

The high oxygen environment of mitochondria indicates that more than 90% of the intracellular O_2_ is consumed by the mitochondria. Most of the ROS produced by cardiomyocytes are generated in the mitochondria. Therefore, we used MitoSOX Red, a fluorescent dye that specifically targets mitochondrial superoxide in living cells, to assess the ROS levels in the mitochondria of WT and KO cardiomyocytes. Compared with WT cardiomyocytes, mitochondrial ROS in KO cardiomyocytes was significantly increased (Fig. 4F and Additional file 1: Fig. S3D), indicating that the mitochondrial oxidative stress level of KO cardiomyocytes was significantly higher.

In addition, the mitochondrial membrane potential of WT and KO cardiomyocytes was measured by immunofluorescence staining and TMRE fluorescence probe. When the mitochondrial function is normal, the mitochondrial membrane potential is higher and the TMRE fluorescent probe emits red fluorescence. When the mitochondrial function was abnormal, the membrane potential decreased, and the fluorescence intensity of TMRE fluorescence was significantly weakened, reflecting the decreased membrane potential. This transformation can be directly used to evaluate the mitochondrial function index [24]. We took the mitochondrial membrane potential level of WT hiPSC-CMs treated with CCCP as the negative experimental control. Immunofluorescence staining showed that the red fluorescence intensity of WT cardiomyocytes was significantly higher than that of KO cardiomyocytes (Fig. 4G). This suggests that the mitochondrial membrane potential of cardiomyocytes was significantly reduced following COX6A2 knockout and mitochondrial function is impaired, which is consistent with the phenomenon of cellular calcium overload. Cellular calcium overload results in a decrease in mitochondrial membrane potential. Ca^2+^ in mitochondria is an important sensitive signal that affects mitochondrial function. Ca^2+^ overload in the mitochondria of cardiomyocytes causes an increase in mitochondrial ROS, thus affecting mitochondrial function.

### COX6A2-knockout cardiomyocytes exhibit reduced energy metabolism

The contraction and relaxation of cardiomyocytes require significant energy [25]. When the myocardial cell energy supply experiences problems, the normal function of the heart muscle cells is affected. Thus, the myocardial cell energy metabolism disorder is considered a key factor for cardiovascular disease. Recent studies have demonstrated that the energy metabolism associated with myocardial cell dysfunction during heart failure plays a key role in the process of development [26].

**Fig. 5:**
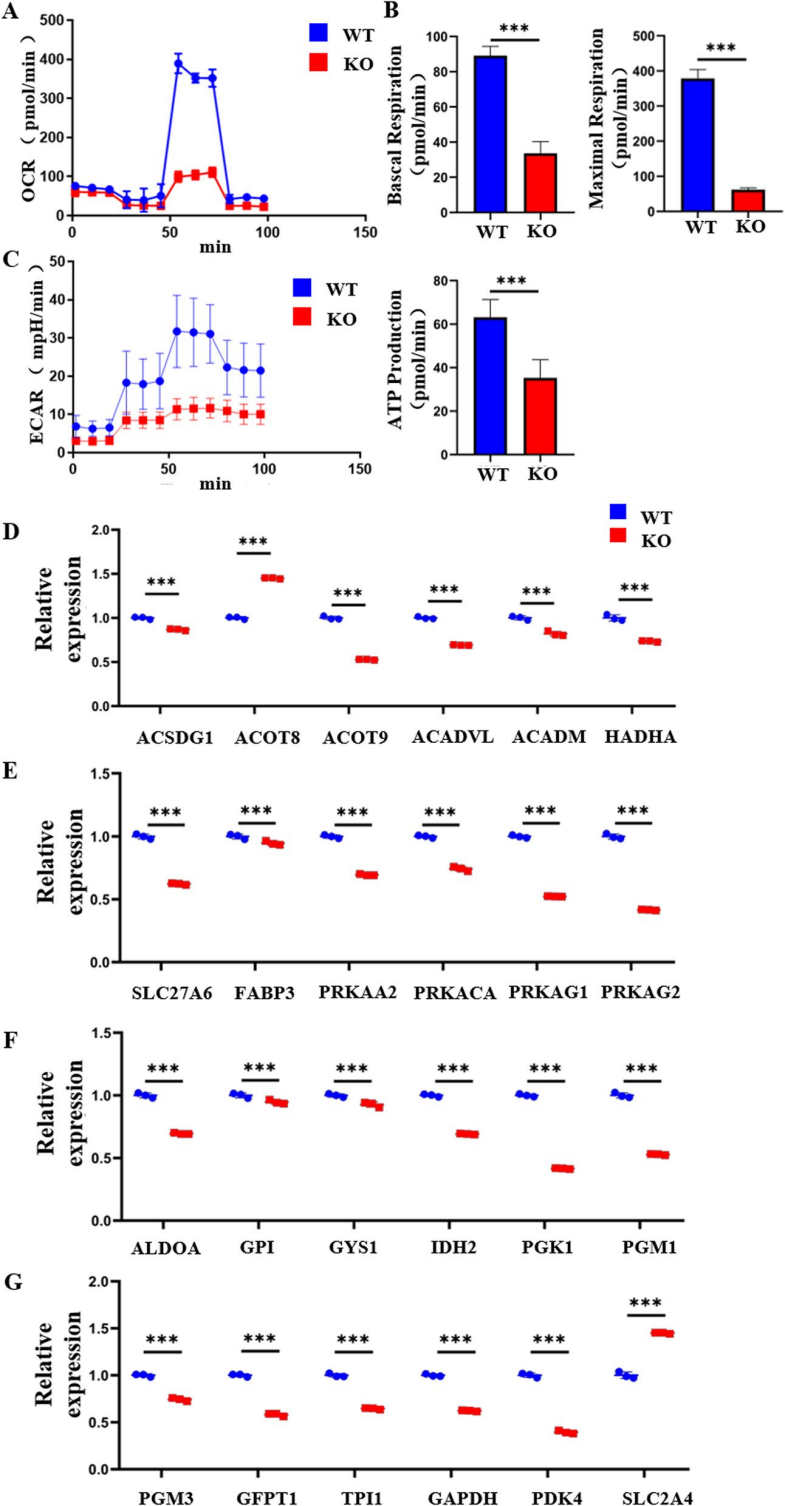
The energy metabolism level of KO hiPSC-CMs was affected. **A** The OCR curves of WT and KO hiPSC-CMs were detected by Seahorse experiments, suggesting that the aerobic respiration level of KO hiPSC-CMs was reduced. **B** Quantitative statistical analysis of basal and maximal respiration levels in WT and KO hiPSC-CMs indicated that basal and maximal respiration levels were decreased in KO hiPSC-CMs. **C** The ECAR curves of WT and KO hiPSC-CMs were detected by Seahorse experiments, suggesting that the level of glycolysis is reduced in KO hiPSC-CMs. **D**, **E** The expression levels of fatty acid metabolism-related genes in WT and KO hiPSC-CMs were detected by qPCR. **F**, **G** The expression levels of genes related to glucose metabolism in WT and KO hiPSC-CMs were detected by qPCR. The results are presented as mean ± SD of 3 independent experiments. ***P < 0.001

ATP in normal cardiomyocytes is primarily produced by mitochondrial oxidative phosphorylation. To measure the level of mitochondrial oxidative phosphorylation in WT and KO cardiomyocytes, the Seahorse XF assay was applied to both groups of cardiomyocytes. The oxygen consumption rate and the extracellular acidification rate (ECAR) were used to assess mitochondrial and glycolytic function, respectively. The results (Fig. 5A) indicated that the basal respiration, maximal respiration, and ATP production of the KO cardiomyocytes were significantly lower compared with that of WT cardiomyocytes (Fig. 5B). The extracellular acidification rate of KO cardiomyocytes was also significantly lower compared with that of WT cardiomyocytes, suggesting that the glycolytic capacity of KO cardiomyocytes was significantly reduced (Fig. 5C). These results indicate that the oxidative phosphorylation and glycolytic capacity of mitochondria are significantly reduced in COX6A2 knockout cells.

Combined with the previous experiments showing that COX6A2 knockout results in mitochondrial dysfunction in cardiomyocytes, we examined changes in the energy metabolism of cardiomyocytes following COX6A2 knockout. Most of the energy supply of cardiomyocytes is contributed by fatty acid and glucose metabolism to produce ATP for energy [27, 28]. Therefore, we measured the expression of genes involved in fatty acid and glucose metabolism in WT and KO cardiomyocytes. Quantitative PCR was used to detect the relative expression of fatty acid metabolism-related genes in WT and KO cardiomyocytes. Twelve genes (ACSDG1, ACOT8, ACOT9, ACADVL, ACADM, HADHA, SLC27A6, FABP3, PRKAA2, PRKACA, PRKAG1, and PRKAG2) that were highly expressed in cardiomyocytes were selected for measurement. The results indicated that the level of fatty acid metabolism was significantly reduced in COX6A2 knockout cardiomyocytes (Fig. 5D, E). Next, we examined the genes involved in glucose metabolism in the two groups of cardiomyocytes. Twelve glucose metabolism pathway-related genes (ALDOA, GPI, GYS1, IDH2, PGK1, PGM1, PGM3, GFPT1, TPI1, GAPDH, PDK4, SLC2A4) that were highly expressed in cardiomyocytes were also selected for detection. The overall trend of glucose metabolism in COX6A2 knockout cardiomyocytes was decreased (Fig. 5F, G). These results indicate that following COX6A2 knockout, KO cardiomyocytes exhibited obvious metabolic disorders in fatty acid and glucose metabolism pathways, and the energy supply of cardiomyocytes is insufficient, which is consistent with the pathological phenotype of myocardial remodeling.

### Abnormal calcium transient in hiPSC-CMs after COX6A2 knockout

**Fig. 6:**
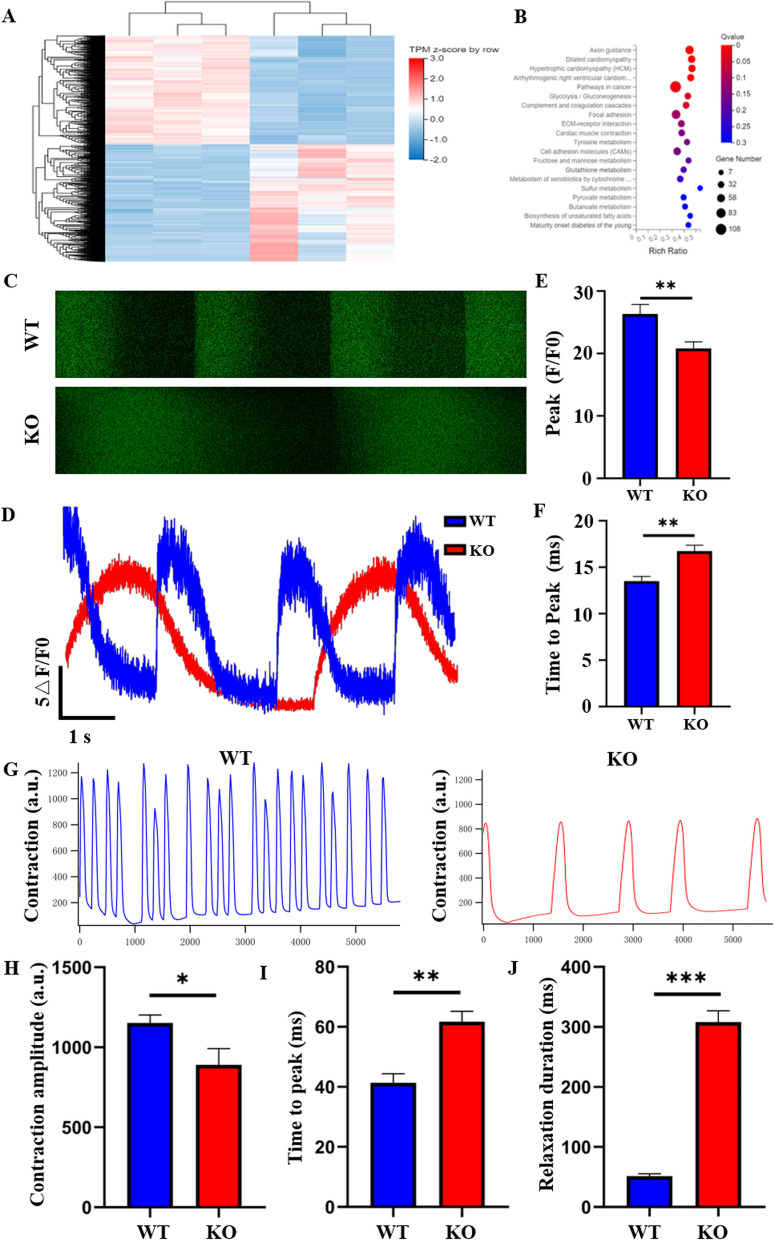
COX6A2 knockout affects calcium activity in cardiomyocytes. **A** RNA-Seq was used to detect differentially expressed genes in WT and KO hiPSC-CMs. **B** KEGG analysis of pathway enrichment changes in KO hiPSC-CMs. **C** Ca^2+^ transient fluorescence patterns of WT and KO hiPSC-CMs were observed by confocal (Fluo-4 AM labeling). **D** Waveform plots of Ca^2+^ transients in WT and KO hiPSC-CMs. **E** Quantitative statistical plots of the Peak of Ca^2+^ transients in WT and KO hiPSC-CMs, which are decreased in KO hiPSC-CMs. **F** Quantitative statistical plots of the Time to Peak of Ca^2+^ transients in WT and KO hiPSC-CMs, which are increased in KO hiPSC-CMs. **G** Waveform plots of contractile force in WT and KO hiPSC-CMs, showing reduced frequency and intensity of contraction in KO hiPSC-CMs. **H** The quantitative statistical plot of contraction in WT and KO hiPSC-CMs showed reduced contractile intensity in KO hiPSC-CMs. **I** In the quantitative statistical plot of the time to peak in WT and KO hiPSC-CMs, the time to peak in KO hiPSC-CMs was longer than that in WT hiPSC-CMs. **J** Quantitative statistical plots of relaxation time in WT and KO hiPSC-CMs showed that KO hiPSC-CMs had significantly longer relaxation times. The results are presented as mean ± SD of 3 independent experiments. *P < 0.05, **P < 0.01, ***P < 0.001

To identify the molecular mechanism of dilated cardiomyopathy-related phenotypes in COX6A2 knockout cardiomyocytes, we used RNA-Seq technology to detect and analyze changes in the gene expression profiles of the two cardiomyocyte groups. A total of 1956 genes were significantly differentially expressed in KO cardiomyocytes compared with WT cardiomyocytes, of which 1111 were significantly up-regulated and 845 genes were significantly down-regulated (Fig. 6A). We performed a KEGG (Kyoto encyclopedia of genes and genomes) analysis on the two groups of WT and KO cardiomyocytes to identify putative differences in pathway activity between the two groups. The results indicated (Fig. 6B) that pathways associated with hypertrophic and dilated cardiomyopathy were significantly enriched in KO cardiomyocytes compared with WT cardiomyocytes. Moreover, energy metabolism pathways were significantly enriched in KO cardiomyocytes, which is consistent with our previous experimental results. In addition, changes in calcium signaling pathways were observed in KO cardiomyocytes, suggesting that calcium activity is affected in COX6A2 KO cardiomyocytes.

Next, we examined the calcium transients in both groups of cardiomyocytes using Fluo-4 AM probes. Compared with WT cardiomyocytes, the peak value of calcium transients in KO cardiomyocytes was significantly reduced and the time to peak time was significantly prolonged (Fig. 6C–F), indicating that there is an obvious barrier to calcium transients in KO cardiomyocytes.

The contractile function of cardiomyocytes is an important indicator of normal function. Therefore, the contractile force level of cardiomyocytes in both groups was evaluated at 40 days (Fig. 6G). Compared with WT cardiomyocytes, the contraction intensity of KO cardiomyocytes was significantly decreased (Fig. 6H). Moreover, the time to peak (Fig. 6I) and relaxation duration (Fig. 6J) were significantly prolonged. In addition, the contraction frequency of KO cardiomyocytes was significantly decreased, suggesting that the heart rate of KO cardiomyocytes was reduced. These results suggest that loss of COX6A2 may cause myocardial dysfunction, which is one reason for the myocardial remodeling phenotype in KO cardiomyocytes.

### Improving energy metabolism rescues the myocardial remodeling phenotype of COX6A2-KO hiPSC-CMs

Previous experiments showed that COX6A2 knockout leads to energy metabolism disorders in cardiomyocytes and fatty acid and glucose metabolism in KO cardiomyocytes are significantly reduced. Trimetazidine (TMZ) was used to improve the energy metabolism of KO cardiomyocytes. Levo-carnitine (L-carnitine, LC) is an important cofactor in the mitochondrial oxidation of fatty acids. It plays an important role in diseases related to metabolic disorders, particularly mitochondria-related diseases. L-carnitine and its esters can improve mitochondrial dysfunction [29]. TMZ decreased the level of the pro-apoptotic protein BAX and increased the expression of Bcl-2. TMZ increased the production of adenosine triphosphate (ATP) and the activity of superoxide dismutase (SOD) induced by myocardial infarction. It also reduced lipid peroxide (LPO), free fatty acid, and nitric oxide (NO) levels in a concentration-dependent manner [30]. L-carnitine primarily promotes fatty acid metabolism and TMZ promotes glycolysis. We examined the concentration gradient of the therapeutic drugs and determined the optimal concentration of LC 200 µmol/L and TMZ 50 µmol/L. LC and TMZ were added to the maintenance medium for the human cardiomyocytes and the solution was changed daily.

**Fig. 7:**
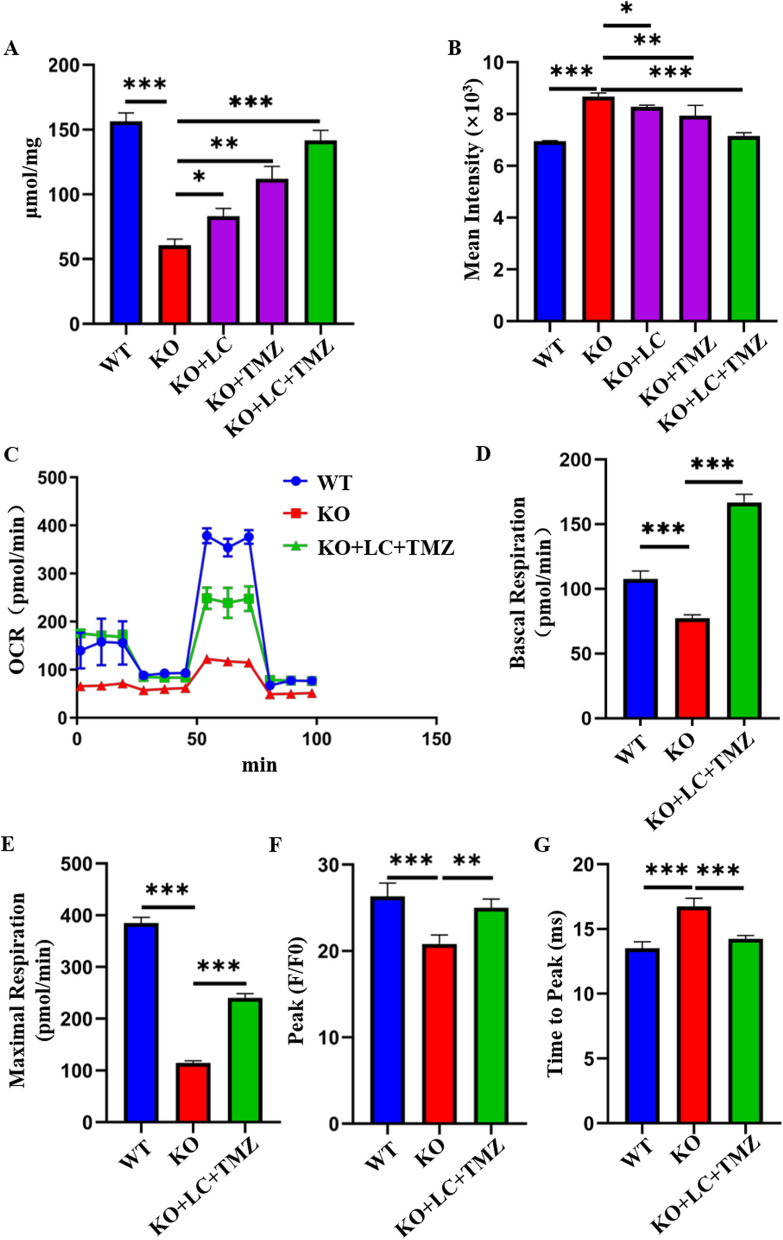
The application of energy-improving drugs can significantly correct the pathological phenotype of KO hiPSC-CMs. **A** The ATP content of WT, KO, KO + LC, KO + TMZ, and KO + LC + TMZ groups was detected, and the results showed that the ATP level of KO hiPSC-CMs increased to varying degrees after different drug treatments. **B** ROS levels in WT, KO, KO + LC, KO + TMZ, and KO + LC + TMZ groups were detected, and the results showed that ROS levels in KO hiPSC-CMs were reduced to varying degrees after different treatments. **C** The OCR curves of WT, KO, and KO + LC + TMZ hiPSC-CMs were detected by Seahorse experiments, the results showed that oxidative phosphorylation was restored in KO hiPSC-CMs after treatment. **D** The basal respiration level of KO hiPSC-CMs was restored after treatment. **E** The maximal respiration level of KO hiPSC-CMs was restored after treatment. **F** The peak level of calcium transient in KO hiPSC-CMs was somewhat restored after treatment. **G** The peak time of calcium transient in KO hiPSC-CMs was significantly decreased after treatment. The results are presented as mean ± SD of 3 independent experiments. *P < 0.05, **P < 0.01, ***P < 0.001

To determine the effects of the two drugs on restoring energy metabolism in KO cardiomyocytes, we first selected a simple method to measure the ATP production of cardiomyocytes following various interventions. An ATP detection kit was used to detect the ATP content of cardiomyocytes in each group following drug administration. The ATP content of KO cardiomyocytes following LC, TMZ, and LC + TMZ treatment was restored to varying degrees, of which LC + TMZ exhibited the greatest effect (Fig. 7A). Combined with the two therapeutic drugs, qPCR was used to determine the effects of LC on fatty acid metabolism and TMZ on glucose metabolism in KO hiPSC-CMs. Both drugs improved energy metabolism in KO cardiomyocytes (Additional file 1: Fig. S5A, B). Similarly, ROS levels in cardiomyocytes were determined after treatment with the three regimens, and the LC + TMZ group exhibited the greatest reduction in ROS (Fig. 7B). Next, the WT, KO, and KO + LC + TMZ myocardial cell groups were evaluated using a Seahorse assay. The results indicated that combination treatment of the KO myocardial cells improved energy metabolism (Fig. 7C–E), indicating that treatment with energy improvement-related drugs is effective. Finally, we measured the calcium transients of cardiomyocytes following treatment, and after drug intervention, the level of calcium transport in cardiomyocytes was restored to a certain extent (Fig. 7F, G). This indicates that improved energy metabolism in COX6A2-deficient cardiomyocytes can restore calcium activity in cardiomyocytes.

## Discussion

Cardiac remodeling refers to the pathological state of abnormal changes in cardiac structure, metabolism, and function caused by myocardial damage and/or increased load during stimulation by various pathogenic factors, which is the initial factor in heart failure caused by various cardiovascular diseases [31]. However, the molecular mechanism of cardiac remodeling remains unclear. Therefore, it is important to elucidate the molecular mechanism of cardiac remodeling, which will lead to new strategies for the clinical prevention and treatment of heart failure. Although there are differences in the mechanisms of cardiac remodeling caused by different etiologies, the disorder of energy metabolism in cardiomyocytes is the final common pathway of cardiac remodeling [2].

Mitochondria are the most important site of myocardial energy production; thus, the maintenance of normal mitochondria function and integrity is important for the maintenance of myocardial tissue structure and physiological function. However, it is difficult to perform high-throughput genomic profiling and pathway selection because primary human cardiomyocytes are difficult to sample and stably maintain in vitro. In addition, commonly used mouse models differ greatly in physiological characteristics from human cardiomyocytes and do not truly reflect the physiological state of the human body [32, 33]. The study of mitochondrial function and cardiac remodeling is limited and only a few proteins, such as DRP1 and OPA1, have been studied in animals [34, 35]. Therefore, new methods are needed to identify the important members of mitochondrial function maintenance and explore their relationship with cardiac remodeling.

hiPSCs are human pluripotent cells with the ability to self-renewal and self-replication. They can proliferate indefinitely in vitro and differentiate into cells representing all germ layers, including cardiomyocytes. hiPSCs are an important type of hiPSCs. Cardiomyocytes differentiated from hiPSCs by specific differentiation protocols in vitro contract spontaneously as in vivo, express sarcoplasmic reticulum proteins and ion channels, and exhibit myocardial-specific action potential and calcium transient characteristics, which can mimic many human cardiomyocyte phenotypes. It has become an important tool for human cardiovascular disease modeling and drug screening [36, 37]. To further study the mechanism of mitochondrial dysfunction and cardiac remodeling, a hiPSC-CM cell model was established. Our results demonstrate that this COX6A2-deficient human cardiomyocyte model could mimic the pathological phenotypes of myocardial remodeling. Because the COX6A2 homozygous knockout cardiomyocyte model is a human cell model, it avoids the species difference between animal and human disease models, and it is phenotypic characteristics and molecular mechanisms are closer to that of human disease. The establishment of the human COX6A2 homozygous knockout cardiomyocyte model facilitates the identification of the molecular mechanism of myocardial remodeling and provides a powerful tool for basic and clinical research.

Considering that COX6A2 functions in the mitochondria, which are the most important cellular energy source, we used a human COX6A2 homozygous knockout cardiac cell model to examine mitochondrial morphology and function as well as energy metabolism before and after COX6A2 knockout. After observing the morphology of cardiomyocytes before and after COX6A2 knockout, we found that the mitochondrial morphology and function of cardiomyocytes were abnormal following COX6A2 knockout. Through our experiment, the expression level of the Drp1 protein in cardiomyocytes was changed after COX6A2 was deleted. Drp1 is activated by two serine sites: p-Drp1 S637 enhances mitochondrial fusion, while p-Drp1 S616 inhibits mitochondrial fusion and promotes mitochondrial classification [38]. This is in contrast to changes in the expression levels of other genes that promote mitochondrial fusion. We speculate that the reason for this may be that COX6A2 does cause calcium overload in cardiomyocytes, which is mainly targeted at Drp1 activation. However, the upregulation of ROS was more significant in COX6A2-KO cardiomyocytes, and the changes in ROS levels were mainly targeted at Mfn genes [39]. Mfn1 and Mfn2 are required for mitochondrial fusion, and Mfn2 can up-regulate ROS levels. In general, the upregulation of genes promoting mitochondrial fusion was more obvious, which was consistent with the mitochondrial morphological changes observed by electron microscopy. Therefore, we can speculate that COX6A2 can indeed lead to increased mitochondrial fusion and decreased division in cardiomyocytes. However, after COX6A2 deletion, ROS accumulation and oxidative stress levels were increased in cardiomyocytes. Mitochondria are the main sites of ROS production in cells, and ROS accumulation directly affects the function of mitochondria in cardiomyocytes. These results indicate that COX6A2 plays an important role in maintaining the normal structure and function of mitochondria.

Using high-throughput transcriptome sequencing analysis, we identified changes in intracellular calcium transport-related pathways in cardiomyocytes. Combined with the results of Ca^2+^ transport experiments in WT and KO cardiomyocytes, we observed that following COX6A2 deletion, Ca^2+^ release and recovery time of KO cardiomyocytes were significantly prolonged, suggesting that the dysfunction of Ca^2+^ transport in KO cardiomyocytes results in Ca^2+^ overload.

When calcium overload occurs in cardiomyocytes, it causes mitochondrial oxidative phosphorylation disorder, a decrease in mitochondrial membrane potential, reduced ATP production, and an increase in ROS levels resulting from the activation of phospholipase and protease in the cytoplasm, which leads to and promotes myocardial cell damage. COX6A2 knockout can cause altered myocardial cell morphology, increased mitochondrial ROS, reduced mitochondrial membrane potential, and mitochondrial calcium overload. Eventually, a "mitochondrial damage mitochondrial energy metabolism disorder-oxidative stress-mitochondrial dysfunction-abnormal myocardial cell calcium transport-mitochondrial damage" negative feedback loop will occur, which may alter the myocardial cell-related pathological phenotypes.

Based on the pathogenic mechanism, we evaluated two drugs with different targets to improve energy metabolism for potential intervention and treatment. Levo-carnitine and TMZ were selected to improve the energy metabolism of COX6A2-knockout cardiomyocytes. L-carnitine primarily promotes fatty acid metabolism and TMZ promotes the glycolytic process. After treatment with L-carnitine and TMZ, the energy metabolism disorder of COX6A2 knockout cardiomyocytes was restored, ATP production was increased, calcium transport was restored, and oxidative stress was reduced. In terms of the degree of phenotypic recovery, the effect of the two-drug combination was superior, and there is likely an additive effect of the combination, which is more conducive to restoring the level of energy metabolism in cardiomyocytes. Therefore, we predict that the combination of multiple drugs may better control the pathological development of myocardial remodeling and achieve the best therapeutic effect.

In summary, we demonstrated that COX6A2 plays an important role in the maintenance of energy metabolism and normal Ca^2+^ activity in cardiomyocytes. This study preliminarily defined the molecular mechanism of myocardial remodeling induced by COX6A2 deletion.

## Conclusions

We developed a COX6A2-deficient cardiac cell model using hiPSCs and a CRISPR/Cas9 system to examine the pathogenic mechanism of myocardial remodeling induced by COX6A2 deletion. This model may be used as an important tool to explore the mechanism of human heart-related diseases, determine the gene-phenotype association, and facilitate the screening of new therapeutic drugs. The human cardiomyocyte model established in this study is a universal technology platform with highly customizable characteristics and broad prospects for clinical translation and application.

### Supplementary Information

**Additional file 1. **Supplementary Data.

**Additional file 2. **Antibodies and Primers.

## Data Availability

All data generated or analyzed during this study are included in this published article. The raw data of RNA-Seq data involved in this manuscript have been uploaded to the GEO database, and the accession number is GSE247580.

## Abbreviations

hiPSC: Human induced pluripotent stem cells
COX6A2: Cytochrome c oxidase subunit 6A2
sgRNA: Single-stranded guide RNA
GA: Glutaric dialdehyde
PFA: Paraformaldehyde
hiPSC-CMs: Human induced pluripotent stem cell-derived cardiomyocytes
WT: Wild-type
PCR: Polymerase chain reaction
TNNT2: Troponin T
α-actinin: Actinin alpha 1
KO: COX6A2^−/−^hiPSC-CMs
LC: L-carnitine
TMZ: Trimetazidine
KEGG: Kyoto encyclopedia of genes and genomes
