# Supplementary material for: COX6A2 deficiency leads to cardiac remodeling in human pluripotent stem cell-derived cardiomyocytes
Source: Stem Cell Res Ther. 2023 Dec 10;14:357. doi: 10.1186/s13287-023-03596-x (PMC10712066; doi:10.1186/s13287-023-03596-x)
Supplement: Supplementary file 1 — Additional file 1. Supplementary Data. [file 13287_2023_3596_MOESM1_ESM.docx]

Supplementary Figure **1.** (A) COX6A2^-/-^hiPSCs grew as clonogenic cell clusters as normal hiPSCs.
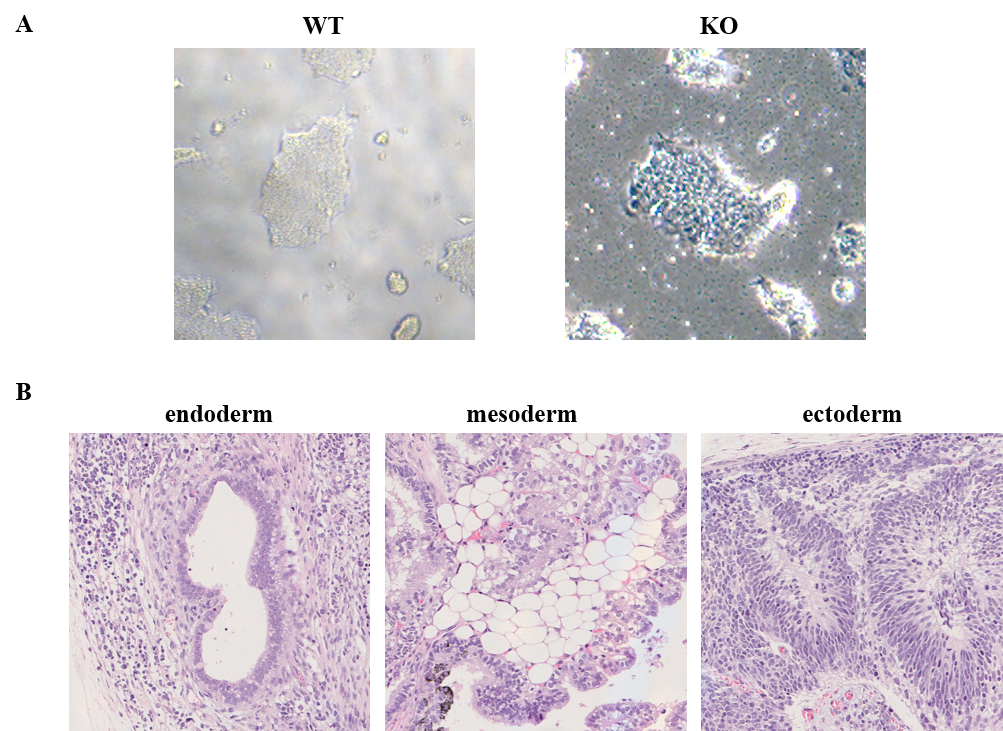
(B) Teratoma assays revealed that COX6A2^-/-^hiPSCs preserved the ability to differentiate into three germ layers.


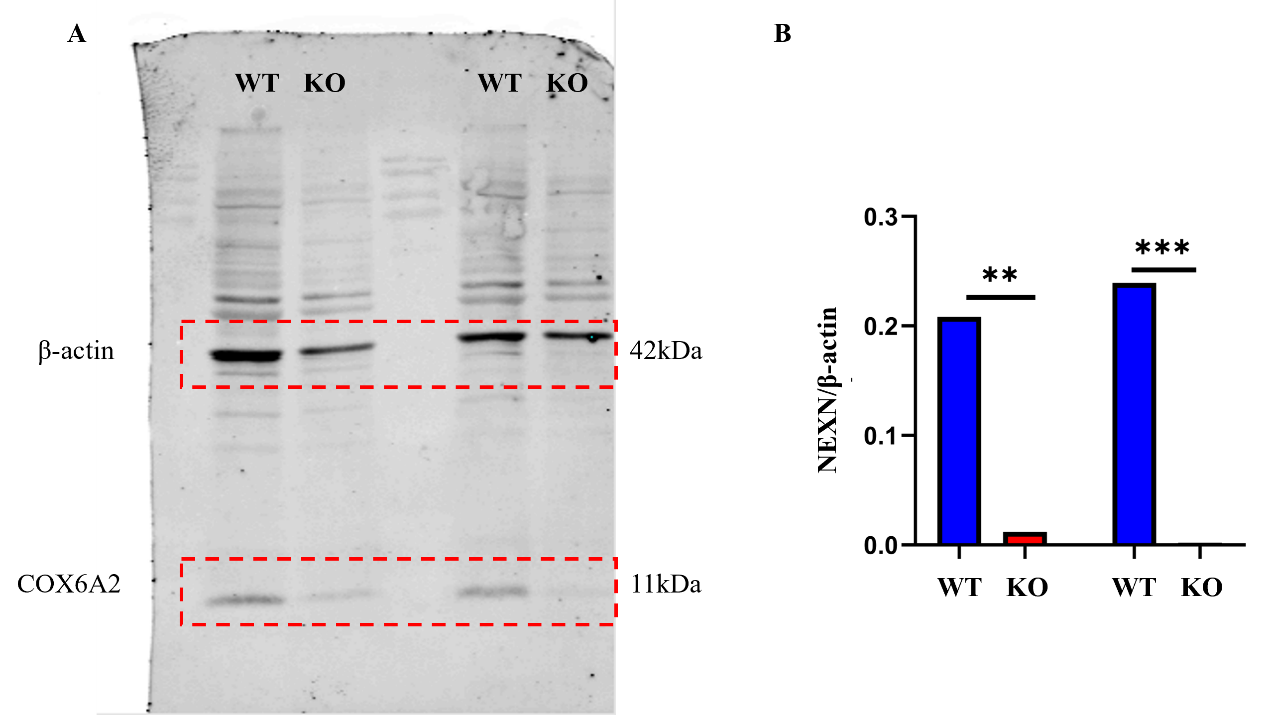


Supplementary Figure **2.** (A) Full-length blots of the expression of COX6A2. (B) The quantification data of the WB result, and the set of data on the right of the statistical chart also represent the WB statistical results of Figure 1D. The results are presented as Mean ± SD of 1 experiment. **P＜0.01, ***P＜0.001.


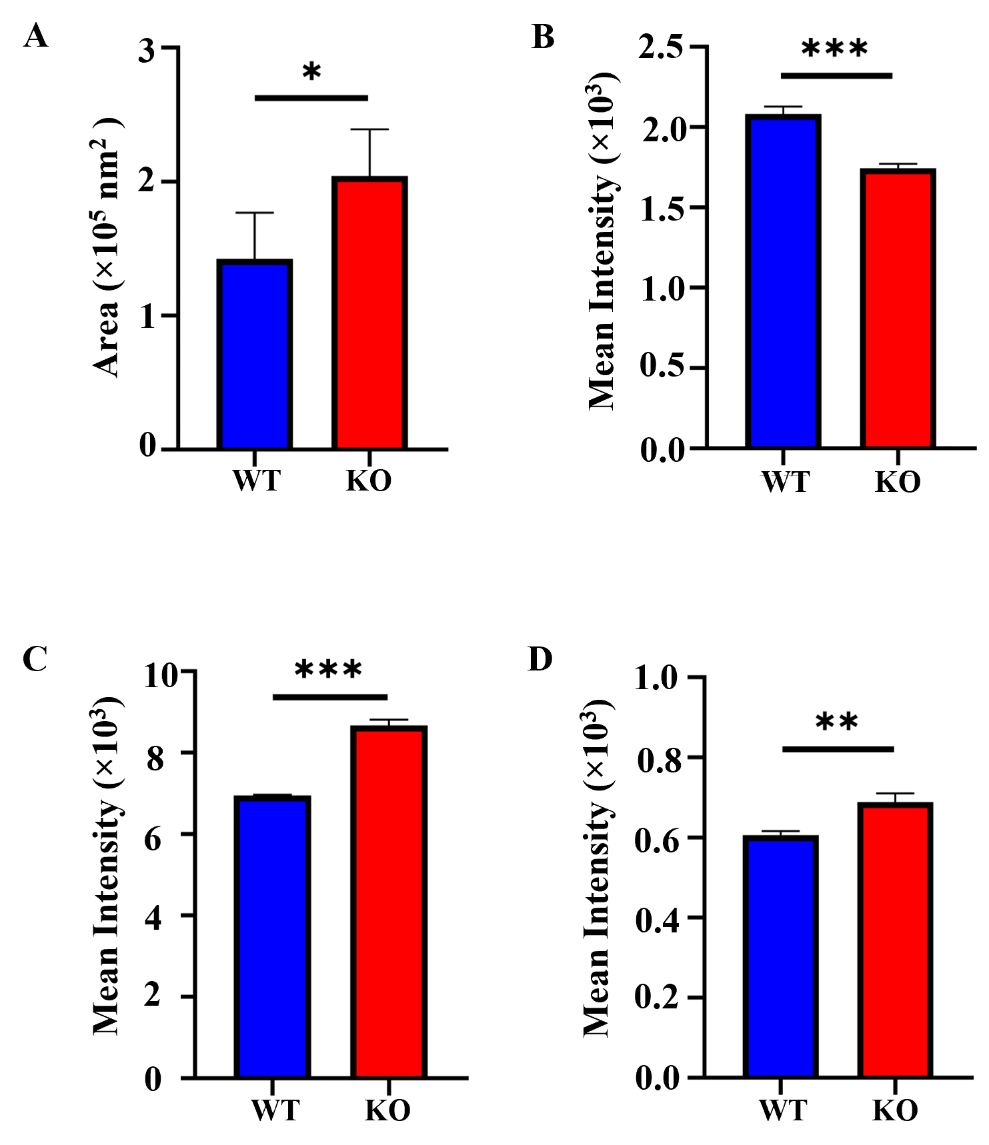
Supplementary Figure 3. (A) Quantitative statistics of mitochondrial surface area measurements in transmission electron microscopy. The results are presented as Mean ± SD of 4 independent experiments. (B)(C)(D) Statistical plots for Mitotracker, ROS, and MitoSOX values quantification of WT and KO hiPSC-CMs. The results are presented as Mean ± SD of 3 independent experiments. *P＜0.05, **P＜0.01, ***P＜0.001.


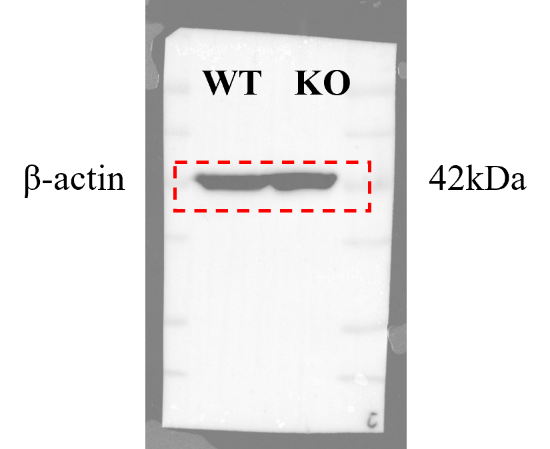

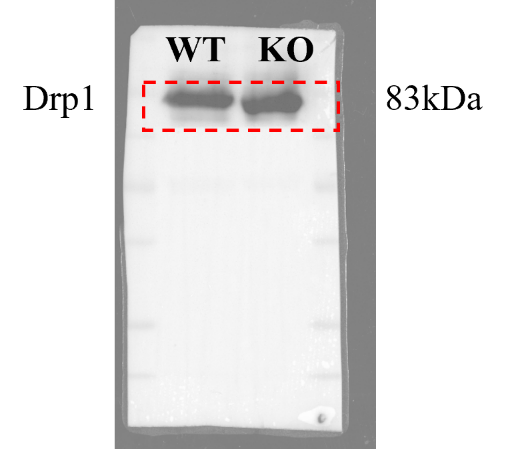

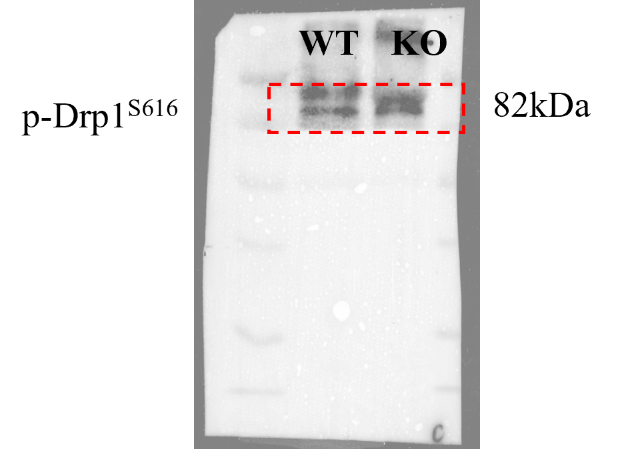

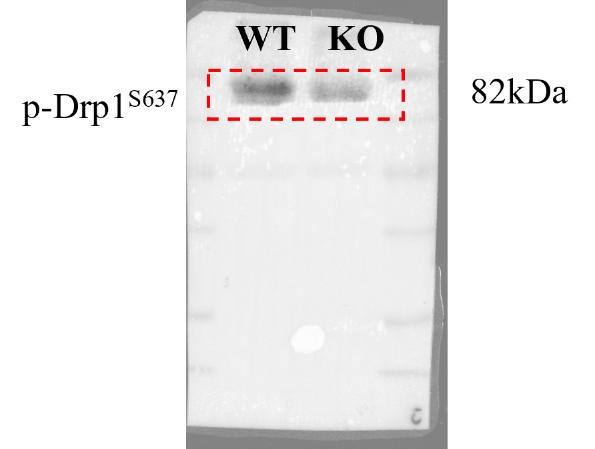

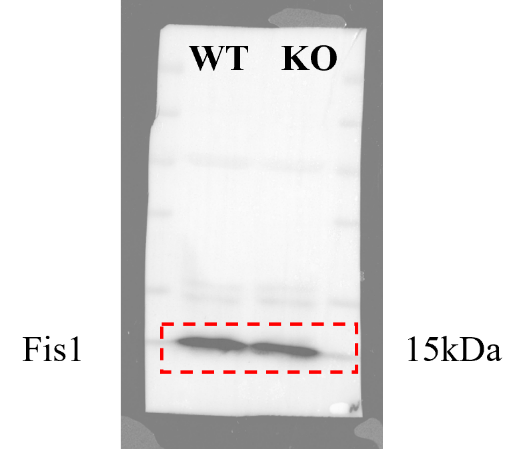

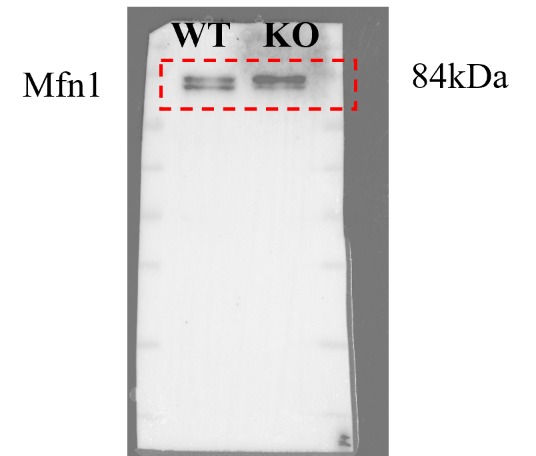

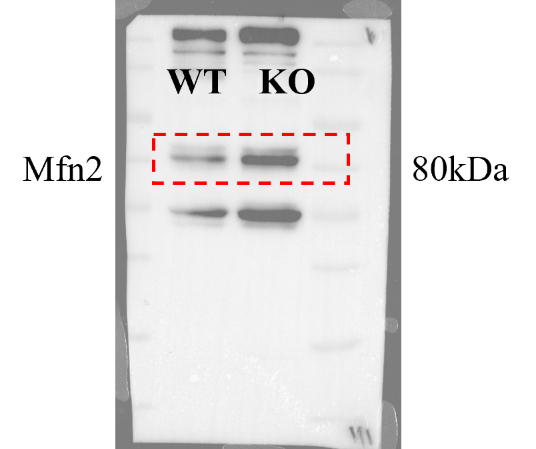


Supplementary Figure 4. Full-length blots of the expression of β-actin, Drp1, p-Drp1S616, p-Drp1S637, Fis1, Mfn1 and Mfn2.

Supplementary Figure **5.**
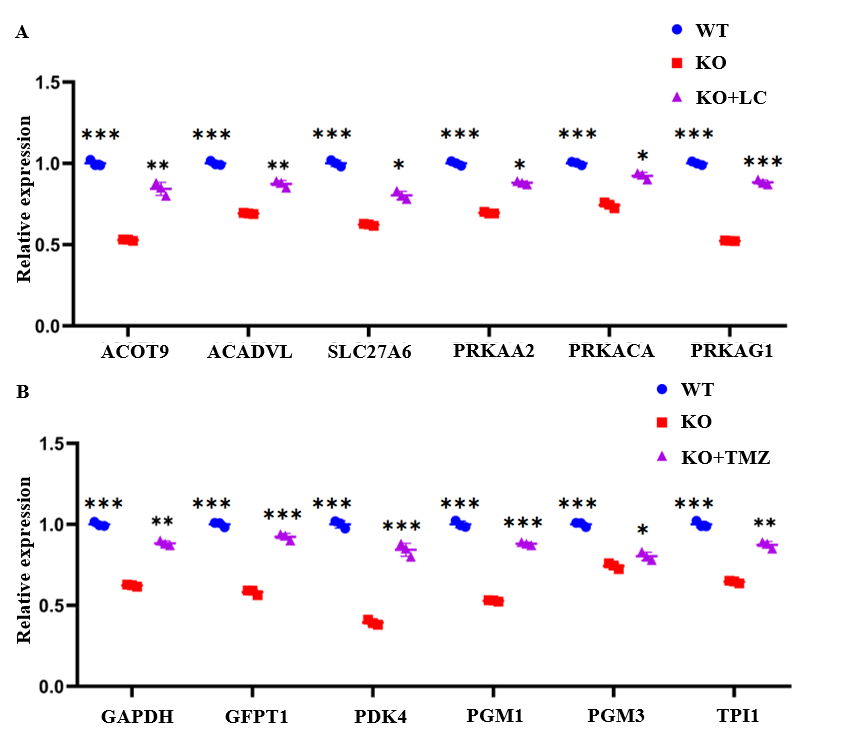
 (A) Q-PCR technique was used to detect the treatment of fatty acid metabolism in KO cardiomyocytes, and it was found that the fatty acid metabolism level of KO hiPSC-CMs was improved after treatment. (B) Q-PCR technique was used to detect the treatment of TMZ on the glucose metabolism level of KO cardiomyocytes, and it was found that the glucose metabolism level of KO hiPSC-CMs was improved after treatment. The results are presented as Mean ± SD of 3 independent experiments. *P＜0.05, **P＜0.01, ***P＜0.001.
