# Supplementary material for: COX6A2 deficiency leads to cardiac remodeling in human pluripotent stem cell-derived cardiomyocytes
Source: Stem Cell Res Ther. 2023 Dec 10;14:357. doi: 10.1186/s13287-023-03596-x (PMC10712066; doi:10.1186/s13287-023-03596-x)
Supplement: Supplementary file 2 — Additional file 2. Antibodies and Primers. [file 13287_2023_3596_MOESM2_ESM.docx]

Table S1. Antibody list

| Name | Description | Manufacturer | |  |
| --- | --- | --- | --- | --- |
| Anti-COX6A2 | Mouse monoclonal | | Abcam | |
| Anti-OCT4 | Rabbit monoclonal | | Biorbyt | |
| Anti-SSEA4 | Mouse monoclonal | | CST | |
| Anti-β-actin | Mouse monoclonal | | Yeasen | |
| Anti-TNNT2 | Mouse monoclonal | | Abcam | |
| Anti-α-actinin | Rabbit polyclonal | | Abcam | |
| Anti-Drp1 | Rabbit monoclona | | Abcam | |
| Phospho-DRP1(Ser616) Antibody | Rabbit | | CST | |
| Phospho-DRP1(Ser637) Antibody | Rabbit | | CST | |
| Anti-Fis1 | Rabbit monoclona | | Abcam | |
| Anti-Mitofusin 1 | Rabbit monoclona | | Abcam | |
| Anti-Mitofusin 2 | Rabbit monoclona | | Abcam | |
| Goat anti-Mouse IgG Alexa Fluor 594 | Goat anti-Mouse | | Invitrogen | |
| Goat anti-Mouse IgG Alexa Fluor 488 | Goat anti-Mouse | | Invitrogen | |
| Goat anti-Rabbit IgG Alexa Fluor 594 | Goat anti-Rabbit | | Invitrogen | |
| Goat anti-Rabbit IgG Alexa Fluor 488 | Goat anti-Rabbit | | Invitrogen | |
| IRDye 800CW Goat anti-Rabbit IgG(H+L) | Goat anti-Rabbit | | LI-COR | |
| IRDye 800CW Goat anti-Mouse IgG(H+L) | Goat anti-Mouse | | LI-COR | |

Table S2. Primer sequences used for q-PCR

| Gene | Direction | Sequence |
| --- | --- | --- |
| SOX2 | Forward | CACATGTCCCAGCACTACCAG |
| SOX2 | Reverse | CACATGTGTGAGAGGGGCAG |
| OCT4 | Forward | CCTGAAGCAGAAGAGGATCACC |
| OCT4 | Reverse | AAAGCGGCAGATGGTCGTTTGG |
| Gene | Direction | Sequence |
| ANP | Forward | ACAATGCCGTGTCCAACGCAGA |
| ANP | Reverse | CTTCATTCGGCTCACTGAGCAC |
| BNP | Forward | TCTGGCTGCTTTGGGAGGAAGA |
| BNP | Reverse | CCTTGTGGAATCAGAAGCAGGTG |
| COX6A2 | Forward | TACCGCCCTGGTAAACTCAAA |
| COX6A2 | Reverse | TACTCTTGGCACAGTTTCCAG |
| GAPDH | Forward | GAAGGTCGGAGTCAACGGATTT |
| GAPDH | Reverse | CTGGAAGATGGTGATGGGATTTC |
| β-actin | Forward | GATCTGGCACCACACCTTCT |
| β-actin | Reverse | GGGGTGTTGAAGGTCTCAAA |
| COL1A1 | Forward | GATTCCCTGGACCTAAAGGTGC |
| COL1A1 | Reverse | AGCCTCTCCATCTTTGCCAGCA |
| COL4A1 | Forward | CCAGGGGTCGGAGAGAAAG |
| COL4A1 | Reverse | GGTCCTGTGCCTATAACAATTCC |
| MYH6 | Forward | TCTCCGACAACGCCTATCAGTAC |
| MYH6 | Reverse | GTCACCTATGGCTGCAATGCT |
| MYH7 | Forward | GGCAAGACAGTGACCGTGAAG |
| MYH7 | Reverse | CGTAGCGATCCTTGAGGTTGTA |
| Cav1.2 | Forward | GCAGGAGTACAAGAACTGTGAGC |
| Cav1.2 | Reverse | CGAAGTAGGTGGAGTTGACCAC |
| RYR2 | Forward | AGAACTTACACACGCGACCTG |
| RYR2 | Reverse | CATCTCTAACCGGACCATACTGC |
| JPH2 | Forward | ACTCTGGCTCCTGGAACTTTG |
| JPH2 | Reverse | GCGCCCCTTGGTCTCTATG |
| SERCA2a | Forward | AAATGGGCAAAGTGTATCGACA |
| SERCA2a | Reverse | CTTGATGACGGAGACAGATTCAC |
| ND-1 | Forward | ATGGCCAACCTCCTACTCCTCATT |
| ND-1 | Reverse | TTATGGCGTCAGCGAAGGGTTGTA |
| ND-2 | Forward | CCATCTTTGCAGGCACACTCATCA |
| ND-2 | Reverse | ATTATGGATGCGGTTGCTTGCGTG |
| ACSBG1 | Forward | CCCCGGAGTGGTTCTTCTC |
| Gene | Direction | Sequence |
| ACSBG1 | Reverse | CATAAGCGATGTACTGGCAGG |
| ACOT8 | Forward | GTGGCCGCCTATATCTCCG |
| ACOT8 | Reverse | CGTGGAACCACATGGAATGG |
| ACOT9 | Forward | AGTTGCGGGAGATAGTAGGAG |
| ACOT9 | Reverse | AGGCAGTCCATCCTGTGATTT |
| ACADVL | Forward | TCAGAGCATCGGTTTCAAAGG |
| ACADVL | Reverse | AGGGCTCGGTTAGACAGAAAG |
| ACADM | Forward | ACAGGGGTTCAGACTGCTATT |
| ACADM | Reverse | TCCTCCGTTGGTTATCCACAT |
| HADHA | Forward | AAATTGACAGCGTATGCCATGA |
| HADHA | Reverse | GCTTTCGCACTTTTTCTTCCACT |
| SLC27A6 | Forward | CTTCTGTCATGGCTAACAGTTCT |
| SLC27A6 | Reverse | AGGTTTCCGAGGTTGTCTTTTG |
| FABP3 | Forward | CATGACCAAGCCTACCACAAT |
| FABP3 | Reverse | CCCCAACTTAAAGCTGATCTCTG |
| PRKAA2 | Forward | GTGAAGATCGGACACTACGTG |
| PRKAA2 | Reverse | CTGCCACTTTATGGCCTGTTA |
| PRKACA | Forward | AGCCCACTTGGATCAGTTTGA |
| PRKACA | Reverse | GTTCCCGGTCTCCTTGTGT |
| PRKAG1 | Forward | ATGAAGTCTCATCGCTGCTATG |
| PRKAG1 | Reverse | ACCGTTAGTCACCAAAGCAAA |
| PRKAG2 | Forward | TGCCCGTTATTGACCCTATCA |
| PRKAG2 | Reverse | CAGGCTTTGGCATATCAGACAT |
| ALDOA | Forward | CAGGGACAAATGGCGAGACTA |
| ALDOA | Reverse | GGGGTGTGTTCCCCAATCTT |
| GPI | Forward | CCGCGTCTGGTATGTCTCC |
| GPI | Reverse | CCTGGGTAGTAAAGGTCTTGGA |
| GYS1 | Forward | CAGCGCGGACCAACAATTTC |
| GYS1 | Reverse | TCCTCCCGAACTTTTCCTTCA |
| IDH2 | Forward | CGCCACTATGCCGACAAAAG |
| IDH2 | Reverse | ACTGCCAGATAATACGGGTCA |
| Gene | Direction | Sequence |
| PGK1 | Forward | GAACAAGGTTAAAGCCGAGCC |
| PGK1 | Reverse | GTGGCAGATTGACTCCTACCA |
| PGM1 | Forward | GATGGGGATCGAAACATGATTCT |
| PGM1 | Reverse | GCTGGAAATACGGAATGCTGAA |
| PGM3 | Forward | GGAGGCAATCAATGACCTGGTG |
| PGM3 | Reverse | CCAAGCTCACTTCATGTGCAAGG |
| GFPT1 | Forward | CCCTCTGTTGATTGGTGTACGG |
| GFPT1 | Reverse | GGAAAAGGCAGGTTGTGCTGTC |
| TPI1 | Forward | ACTGCCTATATCGACTTCGCC |
| TPI1 | Reverse | AAGCCCCATTAGTCACTTTGTAG |
